# Genome characterization of bile-isolated *Shewanella algae* ACCC

**DOI:** 10.1186/s13099-018-0267-4

**Published:** 2018-09-18

**Authors:** Shu-Ying Tseng, Kwong-Chung Tung, Jan-Fang Cheng, Yi-Hsuan Lee, Zong-Yen Wu, Yu-Kai Hong, Shi-Yu Chen, Yao-Ting Huang, Po-Yu Liu

**Affiliations:** 1 Department of Veterinary Medicine, National Chung Hsing University, Taichung, 40227 Taiwan; 2 Department of Energy, Joint Genome Institute, Walnut Creek, CA 94598 USA; 3 Department of Computer Science and Information Engineering, National Chung Cheng University, Chia-Yi, 62102 Taiwan; 4 Department of Nursing, Shu-Zen Junior College of Medicine and Management, Kaohsiung, 82144 Taiwan; 5 Rong Hsing Research Center for Translational Medicine, National Chung Hsing University, Taichung, 40227 Taiwan; 6 Division of Infectious Diseases, Department of Internal Medicine, Taichung Veterans General Hospital, Taichung, 40705 Taiwan

**Keywords:** Shewanella, Cholecystitis, Whole genome sequencing, Colistin, Carbapenem

## Abstract

**Background:**

*Shewanella algae* has been recognized as an emerging human pathogen. However, not much is known about the mechanism of its pathogenesis and its adaptation to a special niche such as the hepatobiliary tract.

**Results:**

In this study, we isolated the *S. algae* ACCC strain from human bile and performed whole genome sequencing. *S. algae* ACCC consists of a circular 4,743,354-bp chromosome with a GC content of 53.08%, within 4080 protein coding sequences. The genome of strain ACCC contains a number of candidate genes which have been reported to be associated with bile adaption, including *htpB, exbBD*, *wecA*, *galU*, *adeFGH* and *phoPQ* regulon.

**Conclusions:**

Our results highlight the association of *S. algae* with a rare disease profile. Further studies are needed to shed light on the evolution of pathogenesis and the niche adaptation of *S. algae*.

**Electronic supplementary material:**

The online version of this article (10.1186/s13099-018-0267-4) contains supplementary material, which is available to authorized users.

## Background

*Shewanella algae* was first described in 1992 from various clinical samples in Japan [1]. Since then, the organism has been identified in aquatic ecosystems worldwide [2]. Studies have also suggested that it thrives in a wide-range of temperatures and sanities [3].

The manifestations of *S. algae* in human infections are protean; although sepsis, intra-abdominal infections, and soft tissue infections are present in the majority of cases [2]. The hepatobiliary tree is one of the most common sites of isolation and a major source of blood stream infection [2, 4]. Reports in Asia have further demonstrated hepatobiliary disease as an important risk factor for shewanellosis [4].

There are very few studies which have examined the pathogenicity of *S. algae*. Its ability for bile salt adaption is likely to be an important factor for its survival and subsequent infection of the hepatobiliary tract. Whole genome sequencing provides opportunities to address the issue [5]. Currently, there are 12 genomes of *S. algae* accessible on NCBI databases (two clinical isolates from respiratory tract and soft tissue and ten environmental isolates). Here, we report the first whole genome sequence of bile-isolated *S. algae* ACCC.

## Methods

### Strain isolation and characterization

The *S. algae* ACCC was isolated from a bile sample of a cholangitis patient and resistant to 5% ox-bile salts. The strain was identified as *S. algae* by the matrix-assisted laser desorption/ionization time-of-flight mass spectrometry (MALDI-TOF MS) technique, along with 16S rRNA sequencing. The primers used for amplification of the 16S rRNA gene were B27F (5′-AGAGTTTGATCCTGGCTCAG-3′) and U1492R (5′-GGTTACCTTGTTACGACTT-3′). The PCR products were subsequently sequenced and compared with the 16S rRNA bacteria sequence database on the NCBI using BLASTn (optimized for megablast) search algorithm [6].

### Library preparation, whole-genome sequence archive, and de novo assembly

The bacterial genomic DNA was extracted from overnight culture of the *S. algae* ACCC using the QIAGEN Genomic-tip 100/G kit and Genomic DNA Buffer Set (QIAGEN, Valencia, CA) according to the manufacturer’s instructions. Qubit dsDNA HS Assay kit and Qubit 2.0 fluorometer (Life Technologies) were used to measure DNA concentration. A total of 2 µg of each DNA sample was used to build indexed PCR-free libraries, using a multiplexed high-throughput sequencing TruSeq DNA Sample Preparation Kit (Illumina, San Diego, CA) according to the manufacturer’s protocols with minor modifications.

The genomic DNA was subjected to whole genome sequencing on an Illumina MiSeq sequencer using the paired-end 2 × 250 bp sequencing protocol and generated 2,927,608 reads. The total read depth was 186-fold coverage, with a mean read length of 301 bp. Read data was filtered using duk (http://duk.sourceforge.net/) (ktrim = r k = 23 mink = 11 hdist = 1). Low quality (Q-score < 10) reads are trimmed and a read is retained if at least 50 bp by FASTQX-toolkit (https://github.com/agordon/fastx_toolkit). Sequencing data were assembled using Velvet v. 1.2.07 and ALLPATHS v. R46652 using 31 bp *k*-mer size (https://github.com/agordon/fastx_toolkit). Sequencing data were assembled using Velvet v. 1.2.07 and ALLPATHS v. R46652.

### Annotation

The annotation of the *S. algae* ACCC was performed using the National Center for Biotechnology Information (NCBI) Prokaryotic Genomes Automatic Annotation Pipeline (PGAAP), in which the prediction was carried out using Glimmer 3.02 [7]. The non-translated genes were predicted using tRNAScan-SE [8], RNAmmer [9], and RFAM [10]. Functional classification of the predicted genes was performed using RPSBLAST program v. 2.2.15 [11] in conjunction with the COGs (Clusters of Orthologous Groups of proteins) databases using an *E*-*value* threshold < 0.001.

### Identification and comparative analysis of virulence genes

The candidate virulence genes in *S. algae* ACCC, MARS14, and C6G3 genomes were separately identified using the Virulence Factors Database (VFDB) [12]. The assembled genome is first aligned against VFDB protein sequences of the full dataset (Set B) using BLASTX under the following criteria: identity > 45%, aligned length > 450 bp, alignment coverage > 95% and *E*-*value* < 1e−45. If multiple virulence factor genes are overlapped at the same locus in the genome, only the best-aligned virulence factor gene is retained. The ACCC-specific virulence genes were further extracted by excluding those also found in MARS14 and C6G3. In addition, putative antibiotic resistance genes in ACCC strain and public available clinical *S. algae* genomes (MARS 14 and YHL) were predicted using ResFinder 3.0 [13].

### Phylogeny reconstruction

We used OrhoANI to compute the ANI values [14], which has 254 citations, because whole-genome ANI now becomes the standard for species identification/confirmation by NCBI during genome submission. The OrthoANI reconstructs the phylogeny in different ways. First, the genome is chopped into pieces of 1020 bp and BLAST is used to compute the ANI of all homologous pairs. Finally, UPGMA is invoked to cluster the species using the ANI as distance metric. In order to further confirm the whole-genome phylogenetic accuracy of OrthoANI, we also reconstruct whole genome phylogeny independently using REALPHY [15], which is based on PhyML to infer the whole-genome phylogeny using maximum likelihood.

### Quality assurance

Genomic DNA used for sequencing was purified from a pure culture taken from a single colony of the *S. algae* ACCC strain. The bacterium was identified as *S. algae* by both biochemical identification and the MALDI-TOF MS technique. The 16S rRNA gene was sequenced and BLAST was performed against the NCBI database, which revealed no potential contamination of the genomic library. The raw read data was filtered using duk, and quality trimmed with FASTQX-toolkit fastqTrimmer.

## Results and discussion

### General Genome Features of *S. algae* ACCC

**Fig. 1:**
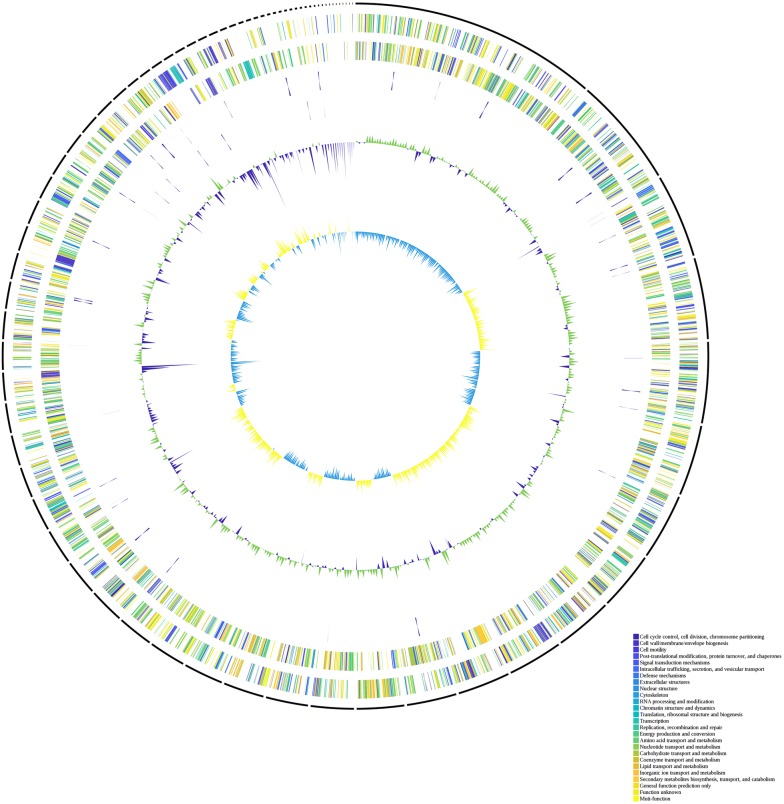
Circular genome map of *S. algae* ACCC. Circles from the outside to inside showing: (1) DNA coordinates; (2, 3) function-based color coded mapping of the CDSs predicted on the forward and reverse strands. Functions are color-code; (4) tRNA genes; (5) rRNA genes; (6) GC plot showing regions above the average (green) and below (violet); (7) GC skew showing regions above average (yellow) and below (light blue)

The final assembled genome consisted of 74 scaffolds with a total size equal to 4,743,354 bp, with a mean G + C content of 53.08%. An illustration of the genomic contents in the genome of ACCC is shown in Fig. 1. The maximum contig size was equal to 589,495 bp, and the N50 size equal to 118,224 bp. The gene annotation included 4080 protein coding sequences (CDSs), 91 tRNA genes and 8 rRNA genes (Additional file 1: Table S1). The distribution of genes into COGs functional categories is shown in Additional file 2: Table S2. No extrachromosomal elements were detected in ACCC.

### Phylogenetic analysis

**Fig. 2:**
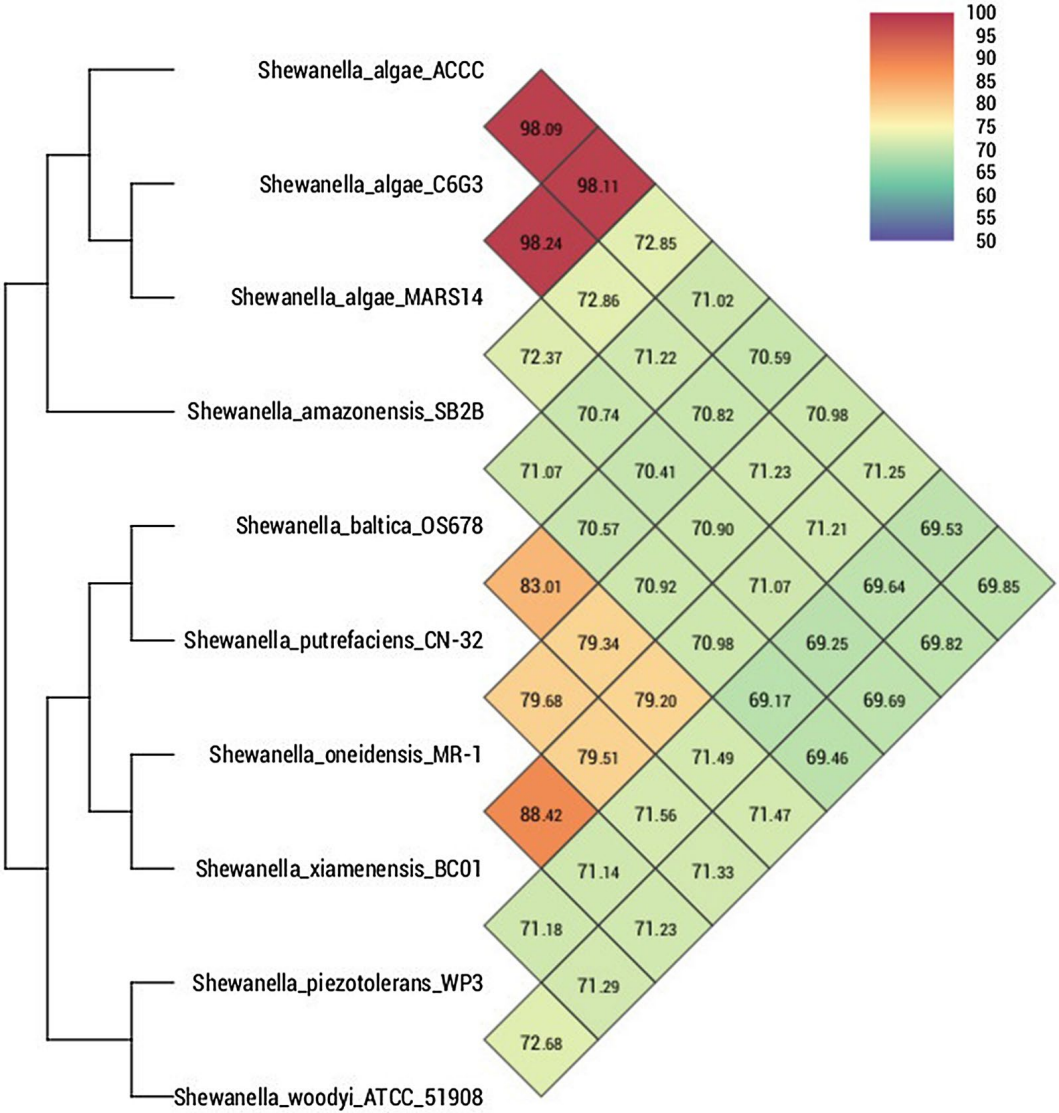
Heat-map and phylogenetic trees based on average nucleotide identity values determined for *S. algae* ACCC and related strains. The values between two strains are given in the junction point of the diagonals departing from each strain

Sequencing of the 16S rRNA gene and a subsequent BLAST-search confirmed the taxonomic status of *S. algae* ACCC (Additional file 3: Figure S1). To further elucidate the phylogenetic relationships, whole genome DNA-sequence-based average nucleotide index analysis was performed and phylogenetic trees were constructed. The dendrogram illustrates that ACCC strain was most closely-related to C6G3 and MARS14, sharing an ANI > 98% (Fig. 2). Independent analysis of Whole-genome phylogeny using REALPHY [15] also confirmed the phylogenetic position of ACCC is indeed most closely-related to C6G2 and MARS14 (Additional file 4: Figure S2).

### Analysis of virulence associated genes

Further screening of the *S. algae* ACCC genome for putative virulence-associated genes was conducted by aligning ORF encoded protein sequences to the virulence factor database. Comparative analysis of ACCC, clinical isolated MARS 14, and environmental isolated C6G2 revealed strain specific genes *mshB* which is associated with MSHA type IV pilus biogenesis. *S. algae* ACCC also contains conservative heat shock protein genes *clpC*, *clpE*, and *clpP*. In addition, the histidine kinase gene *cheA*-*2* was detected and the gene was demonstrated to be essential for chemotaxis [16]. We also predicted *bla*_OXA-55_ in all tested genomes. The clinical significance of this finding warrants further investigation.

### Identification of genes related to bile stress

Bile salts possess potent antimicrobial activity via damage membranes and DNA. To survive in bile and subsequently cause biliary tract infections, bacteria must have intrinsic resistance mechanisms to contend with bile stress [17]. Genomic analysis of *S. algae* ACCC showed the presence of numerous genes which may determine its bile resistance properties, supporting this strain’s pathogenicity in causing cholecystitis. Comparative genomic analysis identified the presence of *htpB* in ACCC but not in clinical isolated MARS 14. *htpB* encoding chaperonin, which has been reported to implicate in bacteria response to bile [18]. We also predicted conservative genes associated with bile adaption. *S. algae* ACCC possessed *exbBD* encoding Ton energy transduction system implicated in the response to bile [19]. We also detected *phoPQ* regulon, *galU*, and *wecA* involved in bile resistance [17]. The gene encoding bile-inducible molecular chaperone DnaK was also identified [17]. The resistance-nodulation-cell division family members have been associated with tolerance to bile salts, multidrug resistance and biofilm formation [20]. Analysis of the genome of strain ACCC revealed the existence of genes encoding resistance-nodulation-cell division pump AdeFGH. Further studies are required to verify its genetic properties, along with the virulence potential, evolution traits for its zoonotic properties and spreading capabilities.

## Additional files

**Additional file 1: Table S1.** General features of *S. algae* ACCC genome.

**Additional file 2: Table S2.** COG functional categories of *S. algae* ACCC genome.

**Additional file 3: Figure S1.** Phylogenetic tree based on 16S rRNA gene sequences showing the phylogenetic position of *Shewanella algae* ACCC.

**Additional file 4: Figure S2.** Phylogenetic tree based on whole-genome sequences showing the phylogenetic position of *Shewanella algae* ACCC.

## Abbreviations

ANI: average nucleotide index
MALDI-TOF: matrix-assisted laser desorption/ionization time-of-flight mass spectrometry
