# Supplementary material for: Genome characterization of bile-isolated Shewanella algae ACCC
Source: Gut Pathog. 2018 Sep 18;10:38. doi: 10.1186/s13099-018-0267-4 (PMC6145196; doi:10.1186/s13099-018-0267-4)
Supplement: Supplementary file 1 — Additional file 1: Table S1. General features of S. algae ACCC genome. [file 13099_2018_267_MOESM1_ESM.docx]

Table S1

General features of *S. algae* ACCC genome.

| Feature | Number |
| --- | --- |
| Genes (total) | 4327 |
| CDS (total) | 4223 |
| Genes (coding) | 4080 |
| CDS (coding) | 4080 |
| Genes (RNA) | 104 |
| rRNAs | 4, 4 (5S, 16S) |
| Complete rRNAs | 4 (5S) |
| Partial rRNAs | 4 (16S) |
| tRNAs | 91 |
| ncRNAs | 5 |
| Pseudo genes (total) | 142 |
