# Supplementary material for: Genome characterization of bile-isolated Shewanella algae ACCC
Source: Gut Pathog. 2018 Sep 18;10:38. doi: 10.1186/s13099-018-0267-4 (PMC6145196; doi:10.1186/s13099-018-0267-4)
Supplement: Supplementary file 2 — Additional file 2: Table S2. COG functional categories of S. algae ACCC genome. [file 13099_2018_267_MOESM2_ESM.docx]

**Table S2**

COG functional categories of *S. algae* ACCC genome.

| #COG class | Description | Count | % |
| --- | --- | --- | --- |
| D | Cell cycle control, cell division, chromosome partitioning | 37 | 0.88 |
| M | Cell wall/membrane/envelope biogenesis | 218 | 5.16 |
| N | Cell motility | 73 | 1.73 |
| O | Post-translational modification, protein turnover, and chaperones | 190 | 4.50 |
| T | Signal transduction mechanisms | 226 | 5.35 |
| U | Intracellular trafficking, secretion, and vesicular transport | 76 | 1.80 |
| V | Defense mechanisms | 92 | 2.18 |
| W | Extracellular structures | 0 | 0.00 |
| Y | Nuclear structure | 0 | 0.00 |
| Z | Cytoskeleton | 0 | 0.00 |
| A | RNA processing and modification | 2 | 0.05 |
| B | Chromatin structure and dynamics | 0 | 0.00 |
| J | Translation, ribosomal structure and biogenesis | 196 | 4.64 |
| K | Transcription | 232 | 5.49 |
| L | Replication, recombination and repair | 266 | 6.30 |
| C | Energy production and conversion | 279 | 6.61 |
| E | Amino acid transport and metabolism | 253 | 5.99 |
| F | Nucleotide transport and metabolism | 85 | 2.01 |
| G | Carbohydrate transport and metabolism | 110 | 2.60 |
| H | Coenzyme transport and metabolism | 135 | 3.20 |
| I | Lipid transport and metabolism | 106 | 2.51 |
| P | Inorganic ion transport and metabolism | 253 | 5.99 |
| Q | Secondary metabolites biosynthesis, transport, and catabolism | 52 | 1.23 |
| R | General function prediction only | 0 | 0.00 |
| S | Function unknown | 1,258 | 29.79 |
| - | Muti-function | 84 | 1.99 |
