# Supplementary material for: Genome characterization of bile-isolated Shewanella algae ACCC
Source: Gut Pathog. 2018 Sep 18;10:38. doi: 10.1186/s13099-018-0267-4 (PMC6145196; doi:10.1186/s13099-018-0267-4)

Figure S1

Phylogenetic tree based on 16S rRNA gene sequences showing the phylogenetic position of *Shewanella algae* ACCC


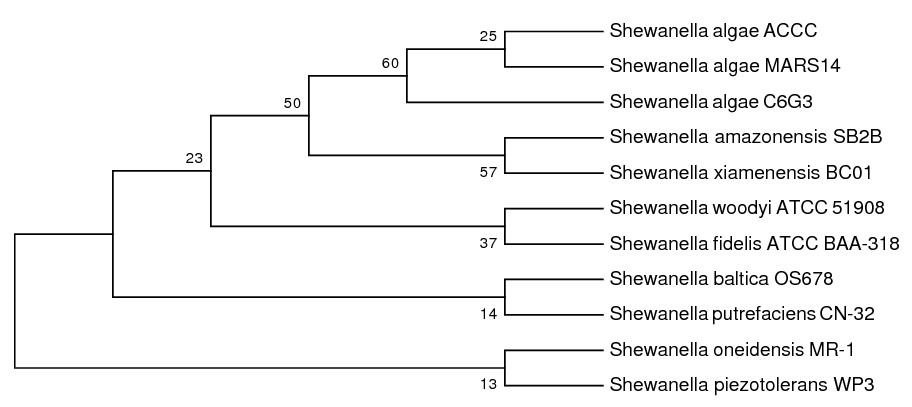

Supplement: Supplementary file 3 — Additional file 3: Figure S1. Phylogenetic tree based on 16S rRNA gene sequences showing the phylogenetic position of Shewanella algae ACCC. [file 13099_2018_267_MOESM3_ESM.docx]
