# Supplementary material for: Genome characterization of bile-isolated Shewanella algae ACCC
Source: Gut Pathog. 2018 Sep 18;10:38. doi: 10.1186/s13099-018-0267-4 (PMC6145196; doi:10.1186/s13099-018-0267-4)

Figure S2

Phylogenetic tree based on whole-genome sequences showing the phylogenetic position of *Shewanella algae* ACCC


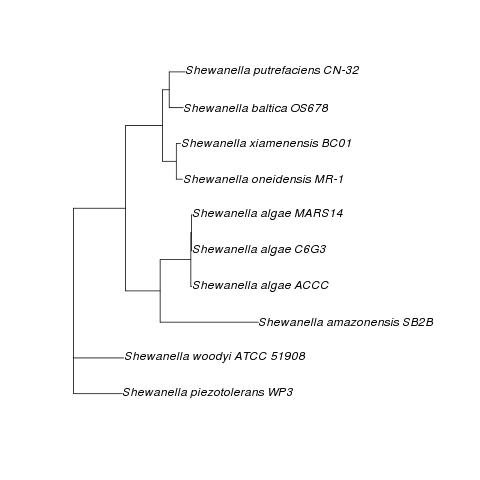

Supplement: Supplementary file 4 — Additional file 4: Figure S2. Phylogenetic tree based on whole-genome sequences showing the phylogenetic position of Shewanella algae ACCC. [file 13099_2018_267_MOESM4_ESM.docx]
